# Supplementary material for: QS21-Initiated Fusion of Liposomal Small Unilamellar Vesicles to Form ALFQ Results in Concentration of Most of the Monophosphoryl Lipid A, QS21, and Cholesterol in Giant Unilamellar Vesicles
Source: Pharmaceutics. 2023 Aug 26;15(9):2212. doi: 10.3390/pharmaceutics15092212 (PMC10537867; doi:10.3390/pharmaceutics15092212)
Supplement: Supplementary file 1 [file pharmaceutics-15-02212-s001.zip › pharmaceutics-2529554-supplementary.pdf]

pharmaceutics

MDPI

Article

QS21-Initiated Fusion of Liposomal Small Unilamellar Vesicles to Form ALFQ Results in Concentration of Most of the Monophosphoryl Lipid A, QS21, and Cholesterolin Giant Unilamellar Vesicles

Erwin G. Abucayon, Mangala Rao, Gary R. Matyas and Carl R. Alving

Centrifugation of ALFQ

The ALFQ suspension containing 22.9 mM bulk phospholipid was initially diluted by 1:10 using Sorensen's phosphate-buffered saline (SPBS). The diluted ALFQ samples (5 mL) were placed in glass centrifuge tubes, and centrifugation was done in a RC5C Sorvall® centrifuged at variable speeds (500 RPM – 8000 RPM) for 10 min at 22 ºC. As shown in Fig. S1A, the ALFQ sample was first centrifuged at 500 RPM. The supernatant was decanted into another clean centrifuged tube, and the pellet was washed 3x with SPBS to remove trapped small vesicles. The supernatant was consecutively centrifuged under the same conditions at 2000 RPM, 3000 RPM, 5000 RPM, 6000 RPM and 8000 RPM. In each centrifugation step, the pellets were washed 3x by SPBS and the final pellets in each step was viewed under light microscope for size characterization. The final pellet was visualized by light microscopy (Fig. S1B). Size characterization of liposomal vesicles in the supernatant and pellets in each centrifugation step was done in bright field at 75x magnification in an Olympus BH2-RFCA microscope equipped with Olympus DP71 camera.

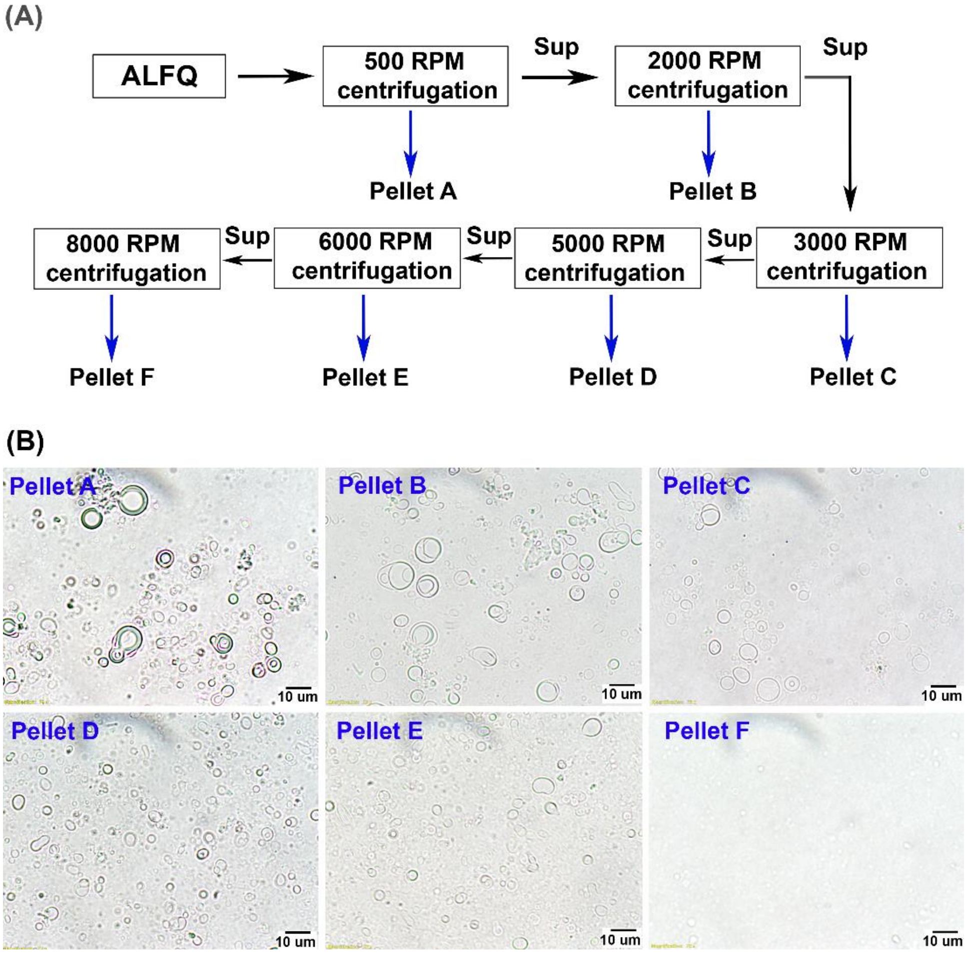

Figure S1. Separation of GUVs using differential centrifugation (DC). (A) Schematic diagram showing the protocol for separating GUVs from ALFQ. ALFQ was first centrifuged at 500 RPM and the pellet was separated. The supernatant was consecutively centrifuged at 2000 RPM, 3000 RPM, 5000 RPM, 6000 RPM, and 8000 RPM. In each centrifugation, the pellets were separated for biophysical characterization. (B) Visualization of the pellets from each centrifugation using phase-contrast light microscopy (75x magnification).

Table S1. UPLC gradient for QS21 quantification.

Time (min)	A (%)	B (%)
–2.0	50	50
0.0	50	50
15.0	0	100
17.0	50	50

Table S2. UPLC gradient for phospholipid quantification.

Time (min)	A (%)	B (%)
-3.0	100	0
6.5	80	20
10.0	50	50
19.0	0	100
20	10	90
26	100	0

Table S3. UPLC gradient for 3D-PHAD® quantification.

Time (min)	A (%)	B (%)
-3.0	100	0
6.5	80	20
10.0	50	50
19.0	0	100
20	5	95
22	5	95

Table S4. UPLC gradient for cholesterol quantification.

Time (min)	A (%)	B (%)
-2.0	100	0
0.0	100	0
6.0	0	100
8.0	5	95
9.0	5	95
